# Mental health distress during the COVID-19 pandemic in Nigeria: Need for psychological intervention

**DOI:** 10.4102/sajpsychiatry.v28i0.1550

**Published:** 2022-01-27

**Authors:** Frances Adiukwu, Margaret Ojeahere, Olufisayo Adesokun, Gbonjubola Babalola

**Affiliations:** 1 Department of Neuropsychiatry, University of Port Harcourt Teaching Hospital, Port Harcourt, Nigeria; 2 Department of Psychiatry, Jos University Teaching Hospital, Jos, Nigeria; 3 Tranquil and Quest Behavioural Health, Lagos, Nigeria

**Keywords:** SARS-CoV-2, psychological distress, Nigeria, worry, mental health needs

## Abstract

**Background:**

The world began to realise the impact of the coronavirus disease 2019 (COVID-19) in January 2020, and since then the number of people infected has exceeded 1 million globally. In less than 1 month following the first reported case in Nigeria, over 180 people had tested positive to the disease. Studies have shown that such rapidly spreading infectious diseases have the potential to create widespread fear, apprehension, panic and anxiety amongst the general public.

**Aim:**

This study aimed at evaluating the impact of information dissemination and public mental healthcare needs during the COVID-19 pandemic in Nigeria. It also hopes to determine if there is an unmet need for telepsychiatry in Nigeria.

**Setting:**

Community-based study covering the North, South and West of Nigeria.

**Methods:**

This was a descriptive cross-sectional study using an on-line survey form via the snowballing sampling method.

**Results:**

Social media was identified as the main source of information concerning COVID-19, and half of the respondents opined that information dissemination was inadequate. Psychological distress was present in 90.5% of the participants and 61.8% admitted that this distress was worsened by fake news and myths concerning COVID-19. However, 53.8% of the participants were willing to access mental healthcare services, with telepsychiatry being the preferred choice.

**Conclusion:**

There is a need to implement a national public mental health service during this emergency. Telepsychiatry has numerous advantages in this context and maybe an opportunity to roll out a novel means of delivering mental healthcare.

## Introduction

The novel coronavirus infection and its associated clinical features, which is reported to have originated from Wuhan, Hubei province of China in December 2019, and officially named by the World Health Organization (WHO) as the coronavirus disease 2019 (COVID-19) caused by the severe acute respiratory syndrome coronavirus 2 (SARS-CoV-2), has created an unprecedented disruptive impact not just on global health, but on the global economy as well.^1^ On realising the global impact of COVID-19, the WHO, on January 30, 2020, declared the disease a Public Health Emergency of International Concern.^2^ Since then, COVID-19 has spread across over 180 countries and territories, including Nigeria, with reported cases as on 02 April 2020 being about 1 004 533 with 51 560 deaths, and 210 583 persons having recovered from the disease.^3^

Nigeria recorded its first case on the 27th of February 2020.^4^ The index case was an expatriate who had flown from Italy via Turkey to Lagos. On 23rd March 2020, Nigeria recorded its first death, a case separate from the index case.^5^ In less than 1 month following the first case in Nigeria, over 180 people had tested positive to a disease previously thought to be alien to Africans.^6^

Despite the relatively low numbers reported, the rapidly escalating nature of its infectivity, and the potential fatality of the disease makes it a huge cause for public concern and worry. Studies have shown that such infectious diseases have the potential to cause widespread fear, apprehension, panic and anxiety amongst the general public.^7,8^

All these may lead to severe psychological distress, sleep disturbances, anxiety, depression, and trauma-related disorders.^9,10^ The associated psychological disturbances are even worse in vulnerable populations such as children, older adults, internally displaced persons, women and people with pre-existing severe mental illnesses.^11,12,13^ Importantly, post-disaster syndromes may not present until weeks and months afterwards.^10^

Sequel to the outbreak, there had been announcements and regular updates on COVID-19 being disseminated by several fora including social media and main stream media. The federal and state governments in Nigeria, the Nigeria Centre for Disease Control (NCDC), federal and state ministries of health, private healthcare facilities, several media and private organisations have been very active in raising awareness in this regard. Much of this information was focused on the role of precautionary measures identified as effective in curbing the spread of the pandemic, and what to do in cases of suspected exposure. Reliable information on addressing the public psychological distress was scarce.

The coronavirus disease 2019 is a novel infectious disease, with an imperfectly understood mode of transmission, poorly elucidated course and potential risk of death, which has left researchers and scientists perplexed regarding treatment modalities.^14^ In order to reduce the rate of transmission, the strictest measures of public health prevention and infection control are being applied. These measures include self-isolation, quarantine, movement restriction and social distancing. They may involve an alteration in usual social practices and have led to the loss of routine pleasurable activities. For example, in Nigeria, identified places where people usually access social support like churches, schools, workplaces and clubs have had their access limited or have been shut down.^15^ This is further worsened by the enforcement of the lockdown measures on the citizenry by the government without considering the mental, social, physical and economic preparedness of individuals and families. In a country with over 40% of its populace living below poverty line,^16^ there is bound to be a resultant breakdown and significant loss of the social support system thus making people susceptible to psychosocial distress and mental health problems.

Having mental health workers playing an active role during an infectious disease outbreak is not a new concept in itself. During the Ebola outbreak in 2014–2016, mental health providers played a significant role in mitigating the mental health problems amongst sufferers, their families and healthcare workers.^17^ However, putting into perspective the widespread, yet incompletely understood, human-to-human transmissibility of COVID-19, and enforced restrictions in mobility, there is an imperative to change the way in which mental healthcare services can be accessed during the pandemic. Telepsychiatry, which allows the provision of mental health assessment and care through the application of telecommunication technology – telephone, social media, and the web – may be a suitable alternative.^18^ It also offers a safe way for patients to connect with their healthcare providers from their homes without increasing the risk of COVID-19 infection to both patient and healthcare provider.^19^

This study aimed to evaluate the effect of information dissemination on psychological distress amongst community dwelling adults across Nigeria. We further sought to evaluate the awareness of mental healthcare needs during the pandemic amongst the study participants and to determine if there is an unmet need for telepsychiatry in Nigeria.

## Methods

This was a descriptive cross-sectional study using an on-line survey form via the snowballing sampling method.

This method was adopted as a necessity in order to maintain social distance from the study participants as well as adhere to the order on movement restriction so as to reduce the spread of COVID-19. The online survey form was distributed using the ‘SurveyMonkey’ online software.^20^ The invitation to participate in the study (in the form of links) was sent to the general population through various ‘WhatsApp’ social media groups through friends, colleagues and acquaintances to allow for a wider distribution. The invitation also had a message which encouraged everyone who came across the survey link to actively forward it to their contacts (snowball effect).

Acceptance to complete the online form was voluntary and submitting the form via the online platform indicated written informed consent.

The respondents who reported themselves as adults (aged 18 years and above), living in Nigeria, were spread over the six regions, with adequate internet access (defined as availability to the use of the internet through personal devices such as mobile phones, tablets and laptop computers) and good literacy level (having spent a minimum of 6 years in formal education).

The study instrument was a semi-structured questionnaire with three domains, namely information on COVID 19, psychological distress, and access to mental healthcare services. The domains were designed in consultation with an expert group and the questions were developed in such a way to capture the presence of psychological distress (i.e. based on symptom descriptions in International Classificatin of Disease 10), mental health needs of the participants and their preferred means of accessing mental healthcare should the need arises. A draft of the questionnaire was piloted amongst 32 respondents and redundant questions were removed.

The final questionnaire containing questions on their awareness, sources of information, experience of distress, and preference for accessing mental healthcare during the COVID-19 pandemic was administered to the respondents. The questions were a mix of closed-ended, multiple choice and Likert-type choices. The last questions were left open-ended in order to examine the broad theme concerning preferred access and possible barriers to telepsychiatry.

The data from this survey was described using descriptive statistics. Figures and tables were used to display the data and the respondents’ comments on the barriers to telepsychiatry were described quasi-qualitatively.

### Ethical considerations

Ethical clearance was obtained from the Research Ethics Committee of the University of Port Harcourt Teaching Hospital, Rivers State, Nigeria: reference number: UPTH/ADM/90/S.II/VOL.XI/924.

## Results

In this study, 243 respondents fully completed the form out of 282 questionnaires returned by the survey platform.

The respondents were between 22 and 68 years of age, with mean age of 40.5 ± 8.3. About half of the respondents (50.2%) were females, 68.2% were married, 98.8% had a tertiary level of education and 93.9% were in some form of paid employment.

### Information on COVID-19 in Nigeria

**FIGURE 1:**
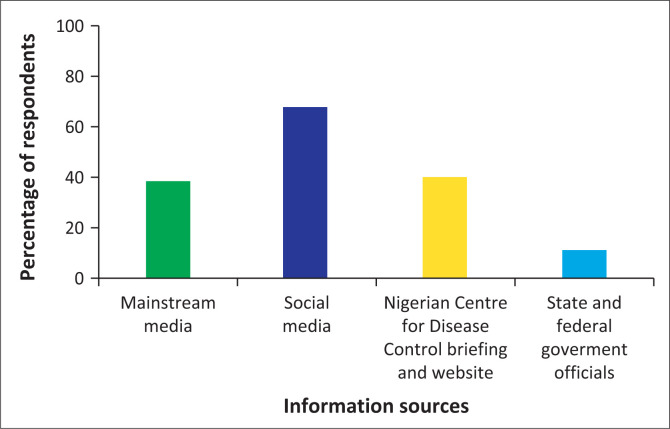
Sources of information on the coronavirus disease 2019 in Nigeria.

In response to the question ‘are you aware of the current COVID-19 pandemic in Nigeria?’, virtually all (99.6%) of the study respondents were aware; however, about half (50.2%) of the participants felt that information dissemination concerning the pandemic was inadequate. Social media was identified as the primary source of information by 68.3% (*n* = 166) of the respondents. (see Figure 1). When asked in a Likert-type question if they felt that myths and fake news about COVID-19 were widespread, 87.2% of the study respondents strongly agreed or agreed. When asked if adequate and up to date information worsened their distress levels, 30.5% of the respondents disagreed, 28.9% agreed whilst 23.0% neither agreed nor disagreed.

### COVID-19 and mental health

Mental healthcare was rated to be an important part of the COVID-19 pandemic response in Nigeria in 95.5% of the participants. However, only 8.6% of the participants felt that the mental health needs of Nigerians were being adequately addressed during this pandemic. When asked to answer yes or no if the pandemic and its containment measures such as lockdown, stay-at-home orders, self-quarantine and movement restrictions would increase psychological distress, a large proportion of the respondents (87.2%) reported that the lockdown and movement restrictions would increase psychological distress, especially fear and worry.

**FIGURE 2:**
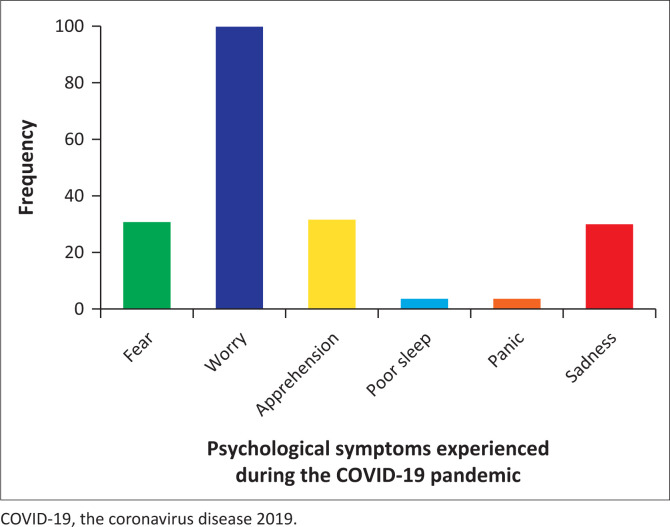
Psychological distress during the COVID-19 pandemic in Nigeria.

About 90.5% of respondents reported experiencing psychological distress by endorsing one or more of fear, worry, apprehension, panic attacks, poor sleep and sadness. Worry (50.0%), fear (15.5%) and apprehension (15.9%) were the most common symptoms reported (see Figure 2). The participants who responded positively to the presence of psychological symptoms were further asked ‘if you experience any of the above symptoms, does fake news and myths about COVID-19 worsen these symptoms?’ more than half (61.8%) of the respondents agreed that myths and fake news about COVID-19 in Nigeria worsened their psychological distress.

### Access preference for mental healthcare services during the COVID-19 pandemic

In response to the question whether it was necessary to have access to mental healthcare during the pandemic 94.4% agreed it was necessary. Almost all (99.4%) of the respondents indicated that they were aware of the two main treatment modalities available (i.e. pharmacotherapy and psychotherapy). When the respondents were asked if they think someone experiencing psychological distress should seek medical care, 89.4% chose ‘yes’. However, only 53.8% of the respondents were willing to access mental healthcare during this period. Of those willing to access care for themselves or their loved ones, when asked to choose one or more of their preferred choice for accessing mental healthcare, the majority indicated that virtual consultations via telecommunication channels would have been their preferred choice of accessing mental healthcare during the pandemic with 56.3% choosing telephone calls and 55.8% instant messages or WhatsApp (see Table 1).

**TABLE 1:** Preferred choices of participants if they were to access mental healthcare during the COVID-19 pandemic in Nigeria (Participants could choose more than one option).

Choice of mental healthcare	Frequency of response	
	*n*	%
Hospital visits	21	12.1
Telephone calls	98	56.3
Instant messaging (SMS, WhatsApp)	97	55.8
Video consultation	5	8.7
Others	16	9.2

SMS, short messaging service.

### Barriers to telepsychiatry

Stigma was identified as a possible barrier in assessing mental healthcare through telepsychiatry. One respondent who reported positive symptoms of worry and fear stated that ‘I do not need it’ regarding assessing virtual mental healthcare. Another barrier to telepsychiatry which we termed ‘poor education’ was identified in 9.2% of the participants. These participants required more information on the modalities of telepsychiatry in order to assess its suitability. They suggested ‘Programmes addressing the topic should also be given adequate publicity on media platforms like Radio and Television at a time like this’ and ‘Proper dissemination of information through radio jingles, television enlightenment programs’.

## Discussion

In this cross-sectional, electronic self-report survey, we found that the respondents were generally aware of the pandemic and about 90% of the respondents reported some form of psychological distress in relation to COVID-19. Although almost all the respondents felt that mental healthcare was necessary, only about half (53.6%) were actually willing to access care for themselves or their loved ones. Most of those willing to seek care preferred to do so through telephonic (56.3%) or instant messaging (55.8%) consultative services.

### Sample characteristics

The sample was limited by the need for internet or data access. This was reflected in the demographics of the study population which was skewed to the relatively higher social economic classes. Despite a reported increase in the total number of internet users in Nigeria, Nigeria still has a generally low internet penetration rate with only about half of its population having access to the internet with a further half of this having daily internet access.^21^ The sample comprised of those who use the internet/ have data and a phone that can support WhatsApp, who received the survey invitation and took out the time to respond.

### Symptoms of psychological distress during the COVID-19 pandemic

More than two-thirds of the respondents in this survey have experienced fear, worry and apprehension since the first identified person infected with novel coronavirus. These findings are suggestive of an increase in anxiety symptoms and probably anxiety disorders in the general population. Anxiety symptoms are frequently reported during large scale disease outbreaks.^22^ A study conducted during the Ebola virus disease (EVD) outbreak reported inability to concentrate and poor sleep as the most occurring symptoms of psychological distress.^23^ Other previous studies have reported widespread apprehension, fear and other anxiety symptoms during an epidemic outbreak.^8,24^ These may lead to the emergence of sleep difficulties, emergent psychological conditions and/or exacerbation of pre-existing symptoms.^9^

Only about one in 10 respondents surveyed feel that their mental health concerns are being addressed during this period. This suggests that there is an unmet need for the provision of mental healthcare services to the populace. During the 2014–2016 Ebola epidemic, mental health providers made important contributions to the care of affected persons, including the front-line healthcare workers.^17^ In the aftermath of the 9/11 catastrophe in the USA, Crisis counselling in particular, was recognised as an effective intervention during the disaster.^11^ It may be worthwhile to consider deploying crisis intervention in this pandemic.

### Roles of disinformation and pandemic containment measures

The fact that 61.7% of the respondents admitted to false information increasing their psychological distress suggests that the high prevalence of fear, worry and apprehension may be at least partly driven by fake news and misinformation. The propagation of myths, fake news and false information concerning COVID-19 has been widespread in Nigeria as in other parts of the world.^25^ Provision of reliable and regular updates by the authorities could be helpful in correcting some of the erroneous messages. There may be a need to explore ways in which sanctions can be imposed on those who carry out these acts by the industry and governments.

The majority of respondents (87.1%) felt that their distress levels would increase with the measures being put in place by the authorities to reduce the spread of COVID-19, such as social distancing, isolation, and movement restriction.

Social connections are protective for mental health, and therefore, the absence of the normal social connectedness, either because of the contagion and/or its containment measures, may lead to an increase in the prevalence of psycho-social difficulties and mental health conditions. Indeed, this is already being reported in some western countries.^26^

### Telepsychiatry in Nigeria during the COVID-19 pandemic

Most of the respondents (89.4%) felt that mental healthcare is important during this pandemic, yet many do not have access to it. On the face of the inadequate number of mental healthcare workers in the country, telepsychiatry may be an effective way to address the issue of access, especially during a contagion.

Interestingly, despite the very high rates of psychological distress reported in this study, the willingness to seek mental healthcare was rather low. About half of the respondents were not willing, despite accessibility to telecommunication platforms, to use it to access mental healthcare. The reasons behind this finding could be related to the stigma generally associated with the use of mental healthcare facilities^27^ (especially in a country like Nigeria where mental health is still highly stigmatised),^28^ the need for personal connectedness (speaking one on one with a therapist rather than virtually) and the lack of adequate information concerning the modalities and scope of telepsychiatry. The unwillingness to seek mental healthcare is a prevailing problem in Nigeria.^29^

However, amongst those willing to access care a favourable acceptability for the use of telepsychiatry service was demonstrated in our study by the fact that the majority of them chose to access mental healthcare service through telecommunication technology.

The demographics of the participants highlights a major challenge to implementing telepsychiatry which is that those living in the rural areas without internet access, those living below the poverty line and those poorly educated may lack adequate access. Several other roadblocks exist – reluctance of the telecommunication providers to collaborate and invest in the health sector, lack of technical expertise by the healthcare providers, fear of litigation and ethical grey areas concerning confidentiality. However, these potential issues could be addressed with specifically tailored interventions. Widespread publicity and innovation in terms of branding may also be important in ensuring optimum uptake. Telepsychiatry in other climes is undergoing rapid changes with a focus on enhancing implementation by navigation of existing bottlenecks in order to provide improved services for patients. For example, some countries are relaxing regulatory requirement to enable mental healthcare providers to offer services during this period of a pandemic.^30^

### Limitations of the study

A number of limitations were noted in this study. Firstly, the cross-sectional nature of the study makes it difficult to ascribe causality to COVID-19 because we could not differentiate between those with prior symptoms. Secondly, carrying out a community-based study involving mental health (a stigmatised topic) was challenging, even without any face-to-face interaction. Despite efforts to reach over a 1000 potential participants through various WhatsApp groups, the number of persons who responded was small. Perhaps there was a reluctance to take part in mental health survey, even with anonymity guaranteed. In addition, the majority of the respondents were employed and relatively well educated which means our findings may not be generalisable to those living in the rural areas without internet access, those living below the poverty line and those poorly educated.

## Conclusion

There is a high prevalence of psychological distress associated with COVID-19 burden, and an unmet need for mental healthcare services to support psychosocial needs of the population during this pandemic. Telepsychiatry may be a way to effectively undertake this intervention. Therefore, the NCDC needs to take this into cognisance in the emergency preparedness, disease prevention and clinical management of COVID-19 in Nigeria.
